# Optimization of the rounded leaf offset table in modeling the multileaf collimator leaf edge in a commercial treatment planning system

**DOI:** 10.1120/jacmp.v15i6.4899

**Published:** 2014-11-08

**Authors:** John R. Rice

**Affiliations:** ^1^ Department of Radiation Oncology Brody School of Medicine, East Carolina University Greenville NC USA

**Keywords:** multileaf collimator, rounded leaf offset, IMRT

## Abstract

An editable rounded leaf offset (RLO) table is provided in the Pinnacle^3^ treatment planning software. Default tables are provided for major linear accelerator manufacturers, but it is not clear how the default table values should be adjusted by the user to optimize agreement between the calculated leaf tip value and the actual measured value. Since we wish for the calculated MLC‐defined field edge to closely match the actual delivered field edge, optimal RLO table values are crucial. This is especially true for IMRT fields containing a large number of segments, since any errors would add together. A method based on the calculated MLC‐defined field edge was developed for optimizing and modifying the default RLO table values. Modified RLO tables were developed and evaluated for both dosimetric and light field‐based MLC leaf calibrations. It was shown, using a Picket Fence type test, that the optimized RLO table better modeled the calculated leaf tip than the Pinnacle^3^ default table. This was demonstrated for both an Elekta Synergy 80‐leaf and a Varian 120‐leaf MLC.

PACS numbers: 87.55.D‐, 87.55.de, 87.55.Qr

## I. INTRODUCTION

Multileaf collimation (MLC) is standard in all modern linear accelerators used to treat patients.^1^, ^2^, ^3^, ^4^, ^5^ The importance of rigorous quality assurance, especially with respect to the MLC and its impact on intensity‐modulated radiation therapy (IMRT) delivery, has been emphasized in the literature.^6^, ^7^, ^8^ As well as quality assurance, the proper modeling of these MLCs in the treatment planning computer is vital to delivering the correct dose to the patient. The Philips Pinnacle^3^ (Philips Medical Systems, Andover, MA) treatment planning software includes default rounded leaf offset (RLO) tables for various vendor's linear accelerators. The RLO table gives the user flexibility in defining the calculated leaf edge. For example, the values would be different depending on whether you calibrate the machine's MLC leaves based on a light field calibration or a dosimetric calibration. Although Philips provides a default table, they do not give the user guidance in how the RLO table may be adjusted to optimize the calculated results with the user's specific machine model. The ideal RLO table values will depend on the user's specific Pinnacle machine model parameters. Nonoptimized RLOs can result in disagreement between the Pinnacle treatment plan and the delivered treatment. This is especially seen in IMRT treatments having a large number of segments with very small areas and low weighting per segment. This paper will show a method of obtaining a user‐defined RLO table. The method is based on calculation by the Pinnacle^3^ treatment planning software and thus is dependent upon the Pinnacle machine's specific modeling parameters. The method is directly applicable if the user defines the MLC leaf position according to the 50% of open‐field intensity at isocenter (dosimetric‐based MLC calibration). If the user defines the MLC leaf position according the light field at isocenter (light field‐based MLC calibration), the method is still applicable using conversion data between the dosimetric and light field calibration values. Such data will be provided for an 80‐leaf Elekta MLC (Elekta Corportation, Stokholm, Sweden). A Picket Fence type test will be used to show that the user‐defined RLO table better matches measured data than the default table.

**Figure 1:**
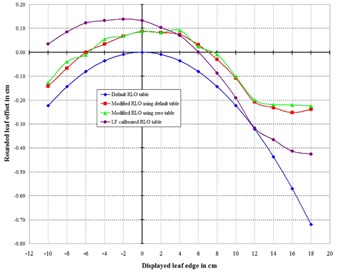
The default Pinnacle RLO table for the 80‐leaf Elekta MLC is shown in blue, the red and green curves are the modified RLO tables for a dosimetric‐based leaf calibration, and the purple curve is the modified RLO table for a light field‐based calibration.

From version 7.4, the Pinnacle treatment planning software has supported the modeling of rounded MLC leaf ends.^(9)^ The graph of the default table in the Pinnacle software for the Elekta 80‐leaf MLC is shown in the blue curve of Fig. 1. For Pinnacle version 9.2, there is only one RLO table for all photon energies of a given machine, and only one table for both sets of leaves. For each leaf position as displayed in Pinnacle, the leaf position for the dose calculation is actually the displayed value plus the value for that position in the RLO table. A positive RLO value moves the leaf towards the opposing leaf bank, a negative value away from the opposing leaf bank. For example, looking at the blue curve of Fig. 1, if the displayed value in Pinnacle of a given leaf is 10.00 cm, the leaf will be moved away from the Y1 leaves by about 2.2 mm (as projected at the plane of isocenter) before the dose is calculated. As can be seen in the default table (Fig. 1), all rounded leaf offsets for the Elekta 80‐leaf MLC are less than or equal to 0, which means the leaves are always moved away from the opposing leaf bank before the dose is calculated.

## II. MATERIALS AND METHODS

**Figure 2:**
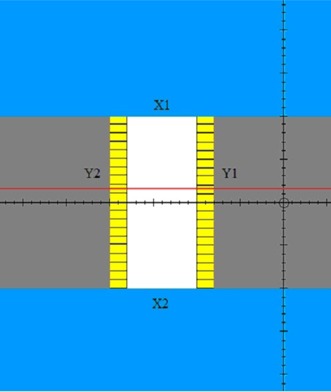
Beam's eye view of Beam 1. The open part of the field is shown in white.

**Figure 3:**
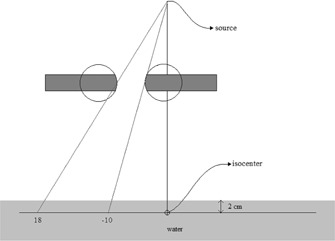
Side view of Beam 1 whose beam's eye view is shown in Fig. 2. The above lines passing through the rounded leaf tips lie along the center of the Y1–19 and Y2–19 leaves of Fig. 2. Each leaf shows a circle whose radius is equal to the rounded leaf radius. Calculated planar doses were obtained through the horizontal line passing through isocenter.

**Figure 4:**
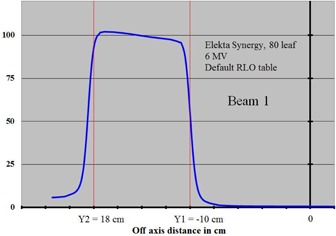
Profile of Beam 1 through leaf 19 using the default Pinnacle RLO table.

A 6 MV beam was defined in Pinnacle as shown in the beam's eye view in Fig. 2. This beam was for an Elekta Synergy 80‐leaf MLC machine. The side view of this beam is shown in Fig. 3. The beam is normally incident on a water phantom cube whose side measures 40 cm. The SSD was set to 98 cm. A planar dose was calculated in the plane containing the isocenter at a depth of 2.0 cm. This planar‐dose plane is shown in Fig. 3 as the black horizontal line in the water. The planar dose had a resolution of 0.5 mm and its lateral dimensions were large enough to encompass the open part of the field. Figure 2 is a beam's eye view of this beam. As shown in Fig. 2, both X jaws, X1 and X2, were set in Pinnacle to 10.0 cm. All Y2 MLC leaves were set in Pinnacle to 18.0 cm and all Y1 leaves to −10.0cm. The Y1 and Y2 backup jaws were set at 2.0 cm behind the MLC leaves so that the Y field edges within the open part of the field were completely defined by the leaves. The open part of the field in Fig. 2 is shown in white, the X jaws in blue, and the backup Y jaws in gray. The default RLO table was selected for the Elekta 80‐leaf MLC (40 Y1 and 40 Y2 leaves with a 1.0 cm leaf width at isocenter) and the dose was calculated. The calculated Pinnacle planar dose file is a simple ASCII text file and was imported into a Quattro Pro (Corel Corporation, Ottawa, ON) worksheet where a single profile was extracted; the line along this profile is shown in red in Fig. 2. This line is taken through the middle of leaf pair 19. The profile for this beam is shown in Fig. 4. This profile is normalized to 100 at the center of the open part of the field. The calculated field edge on the left as defined by the 50% intensity (for the Y2 leaves) and on the right (for the Y1 leaves) were recorded.

The above procedure was repeated with one change, the entire open field as seen in the beam's eye view of Fig. 2 was shifted to the right by 2.0 cm. The X1 and X2 values remained the same at 10.0 cm, but the Y2 MLC leaves were set to 16.0 cm and the Y1 leaves to −8.0cm. The backup jaws were also shifted to the right to always maintain a distance of 2.0 cm behind the Y1 and Y2 leaves. The planar dose was again computed and the profile extracted, as with the first beam. As in the first beam, the calculated left and right field boarder values as defined by the 50% intensity were recorded. Thirteen more beams were calculated in this way, each time shifting the Y1 and Y2 leaves and backup jaws to the right in 2 cm increments. This resulted in 15 Y2 and Y1 calculated field edges at the values corresponding the Pinnacle displayed leaf positions of 18, 16, 14, 12, 10, 8, 6, 4, 2, 0, −2,−4,−6,−8, and −10cm. It must be remembered that all of these calculated field‐edge values were calculated using the default Pinnacle RLO table for an 80‐leaf Elekta MLC. The difference between the calculated field‐edge value and the Pinnacle displayed leaf value for a given position (say Y1=16.0) is the amount by which the default RLO table must be adjusted at that position. This is assuming that a dosimetric calibration of the MLC leaves is being used, as was the case with the author (dosimetric calibration was performed using Elekta software and verified using measurements in a water tank). If one is not using a dosimetric‐based calibration but a light‐based one, the above method is still applicable by utilizing conversion data between these two calibration methods. The following steps were used to measure these conversion data for an Elekta Synergy 80‐leaf MLC.

1. The MLC leaves were calibrated on the linear accelerator using a dosimetric‐based calibration. This was performed with the 6 MV energy. The machine was adjusted until the difference between the displayed leaf position and the radiation‐defined field edge was less than 0.3 mm for leaf positions from −10cm through 18 cm.
2. Graph paper was placed on the treatment table with the table top at isocenter. The crosshairs of the field were aligned with the axes of the graph paper. It was ensured before conducting this experiment that the crosshairs had a run‐out of less than 0.5 mm. The light field of the field shown in Fig. 3 was projected onto the paper. In this field, Y2=18cm and Y1=−10cm, and both the Y1 and Y2 field edges are completely defined by the MLC leaves. It could be seen that the light field edge for both the Y1 and Y2 side were not aligned at −10cm and 18 cm, respectively, as expected.
3. For the Elekta Synergy linear accelerator, the leaves cannot be moved by entering values directly in mm or cm. One must adjust a value referred to as “Y1 cal offset” or “Y2 cal offset” for the Y1 or Y2 leaves, respectively. These offset values are unit‐less potentiometer values. Adjusting the Y1 cal offset value moves all the Y1 leaves by the same amount (the entire Y1 leaf bank moves) and the same holds for the Y2 cal offset and the Y2 leaves. With the graph paper in place, these values were adjusted for each leaf bank until it could be seen that the light field edge aligned with Y2=18cm and Y1=−10cm, as shown in Fig. 4. The changes in Y1 and Y2 cal offset values for each leaf position were then converted to changes in distance in mm.
4. The data from Step 3 were plotted as a function of displayed leaf position. A linear least squares fit was performed on the data, and the slope and y‐intercept were recorded.

**Figure 5:**
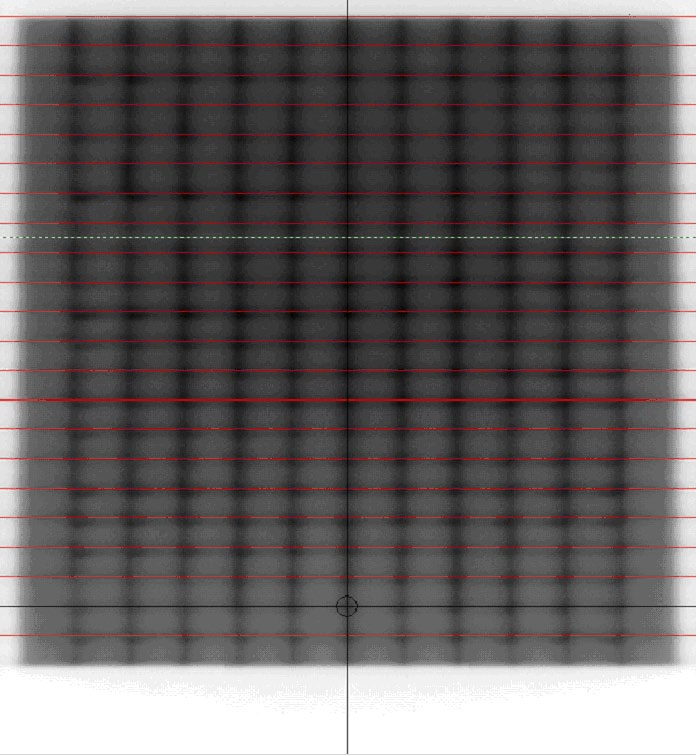
A Picket Fence test for the Elekta 80‐leaf MLC using a 2 mm gap. The “posts” of the test are spaced 2 cm apart. The circle at the bottom of the figure shows the location of isocenter. The red lines show the divisions between the individual MLC leaves. The dashed line is through leaf number 8.

A Picket Fence type test (Fig. 5) was used to test the accuracy of the modified RLO table. A field similar to that shown in Fig. 2 was used, except that the distance between the Y1 and Y2 leaf tips was 2.2 cm instead of 8.0 cm as in Fig. 3. The X1 jaw was set to 20.0 cm and the X2 jaw to 2.0 cm. This produced a field 2.2 cm wide and 22.0 cm in height. The left and right field edges were defined by the MLC leaves with the backup jaws at 2.0 cm behind the leaves. Setting Y2=12.1cm and Y1=−9.9cm, a film was exposed (GAFCHROMIC film, International Specialty Products, Wayne, NJ) producing a strip. The field was then shifted to the right by 2.0 cm so that the first field overlapped the second by 2 mm. This produced a dark, Picket Fence “post” at the overlap. The field was again shifted to the right to produce another 2 mm overlap and the film exposed. This continued until Y2=−9.9cm and Y1=12.1cm. The final result was a film with 11 “posts.” The Picket Fence test was repeated to produce another film, this time using a strip gap of 3 mm. Pinnacle planar doses using both the default RLO table and the modified one were calculated with the same field geometry used for the Picket Fence test on the Elekta Synergy. Planar doses were calculated for both the 2 and 3 mm overlaps.

## III. RESULTS & DISCUSSION

Referring to Fig. 4, it can be seen immediately that, although the Y2 leaves defining the left edge of the field were set to 18.0 cm, the Pinnacle‐calculated left field border as defined by the 50% intensity is greater than 18.0 cm. Likewise, although the Y1 leaves defining the right edge of the field were set to −10.0cm, the Pinnacle‐calculated right field boarder lay slightly to the right of the Y1=−10.0 line of Fig. 4. The Pinnacle‐calculated left field‐edge value for this beam had a value of 18.4682 cm. This value is shown at the bottom of the second column of Table 1. The Pinnacle calculated right field‐edge value was −9.9065 and is shown at the top of the third column of Table 1. This profile analysis was completed for the other 14 fields and the results are shown in the second and third columns of Table 1. The shift necessary to the Y2 leaf at Y=18cm will then be 18.4682–18=0.4682 and this will be added to the default RLO table value at Y=18 which is −0.7198 (see bottom of column 4 in Table 1). The modified Y2 value for Y=18 is then −0.7198+0.4682=−0.2516 and is shown at the bottom of column 5. The modified Y1 value for Y=18 is −0.2262 as seen at the bottom of column 5. Since Pinnacle does not allow a separate RLO table for each of the Y1 and Y2 leaf banks, the average was taken: (−0.2516+−0.2262)/2=−0.2398. The same procedure was followed for each of the other 14 rows of Table 1, resulting in the last column which is the final modified RLO table. These data are shown graphed as the green line of Fig. 1.

**Table 1:** Results of the 15 beams using the default RLO table for the Elekta 80‐leaf MLC. The last column on the right shows the data plotted in green in Fig. 1.

*Displayed Leaf Position (cm)*	*Y2 Leaf Edge (cm)*	*Y1 Leaf Edge (cm)*	*RLO Values (cm)*	*Y2 Modified RLO (cm)*	*Y1 Modified RLO (cm)*	*Av. of Y1 and Y2 Modified RLO (cm)*
−10.0	−9.9302	−9.9065	−0.2234	−0.1536	−0.1299	−0.1418
−8.0	−7.9388	−7.9064	−0.1431	−0.0819	−0.0495	−0.0657
−6.0	−5.9343	−5.9059	−0.0805	−0.0148	0.0136	−0.0006
−4.0	−3.9435	−3.9166	−0.0358	0.0207	0.0476	0.0342
−2.0	−1.9355	−1.9134	−0.0090	0.0555	0.0776	0.0665
0.0	0.0726	0.1006	0.0000	0.0726	0.1006	0.0866
2.0	2.0745	2.1085	−0.0090	0.0655	0.0995	0.0825
4.0	4.0879	4.1325	−0.0358	0.0521	0.0967	0.0744
6.0	6.0881	6.1366	−0.0805	0.0076	0.0561	0.0318
8.0	8.0961	8.1309	−0.1431	−0.0470	−0.0122	−0.0296
10.0	10.0939	10.1371	−0.2234	−0.1295	−0.0863	−0.1079
12.0	12.0988	12.1284	−0.3213	−0.2225	−0.1929	−0.2077
14.0	14.1932	14.2168	−0.4368	−0.2436	−0.2200	−0.2318
16.0	16.3061	16.3289	−0.5697	−0.2636	−0.2408	−0.2522
18.0	18.4682	18.4936	−0.7198	−0.2516	−0.2262	−0.2389

To check the consistency of this modified RLO table, the procedure described in the Methods section was again followed except, this time, an RLO table was used having a value of 0 for each leaf position. All 15 profiles were calculated and processed as described. The data for these 15 profiles are shown in Table 2. The RLO table with zero for each leaf position is seen in column 4 of Table 2. The calculated field edges in columns 2 and 3, as expected, were found to be different from those of Table 1. The modified RLO table, shown in the last column of Table 2, is shown graphed as the red line in Fig. 1 and can be seen to be statistically the same as the green curve, proving that the two methods yield the same result, as expected.

**Table 2:** Results of the 15 beams using a RLO value of 0 for all positions. The last column on the right shows the data plotted in red in Fig. 1.

*Displayed Leaf Position (cm)*	*Y2 Leaf Edge (cm)*	*Y1 Leaf Edge (cm)*	*RLO Values (cm)*	*Y2 Modified RLO (cm)*	*Y1 Modified RLO (cm)*	*Av. of Y1 and Y2 Modified RLO (cm)*
−10.0	−10.1397	−10.1124	0.0	−0.1397	−0.1124	−0.1260
−8.0	−8.0487	−8.0291	0.0	−0.0487	−0.0291	−0.0389
−6.0	−6.0261	−5.9951	0.0	−0.0261	0.0049	−0.0106
−4.0	−3.9558	−3.9368	0.0	0.0442	0.0632	0.0537
−2.0	−1.9476	−1.9164	0.0	0.0524	0.0836	0.0680
0.0	0.0739	0.1028	0.0	0.0739	0.1028	0.0884
2.0	2.0619	2.1036	0.0	0.0619	0.1036	0.0828
4.0	4.0704	4.1149	0.0	0.0704	0.1149	0.0926
6.0	6.0046	6.0446	0.0	0.0046	0.0446	0.0246
8.0	7.9748	8.0077	0.0	−0.0252	0.0077	−0.0087
10.0	9.8820	9.9144	0.0	−0.1180	−0.0856	−0.1018
12.0	11.7838	11.8183	0.0	−0.2162	−0.1817	−0.1989
14.0	13.7693	13.7939	0.0	−0.2307	−0.2061	−0.2184
16.0	15.7670	15.7940	0.0	−0.2330	−0.2060	−0.2195
18.0	17.7646	17.7863	0.0	−0.2354	−0.2137	−0.2246

A modified RLO table using the average of the values of the last column of Tables 1, 2 (the average of the red and green curves of Fig. 1) was created in Pinnacle and the same procedure described in the Methods section was followed to generate 15 beams with 15 profiles. The data were processed as before, obtaining data like those in columns 2 and 3 of Table 2. In this case, the difference between the Pinnacle‐calculated field edge and the displayed leaf position (column 1 of Table 2) was less than 0.1 mm for each of the 15 leaf positions from −10 to 18 cm, showing that the modified Pinnacle RLO table produced the correct results.

**Figure 6:**
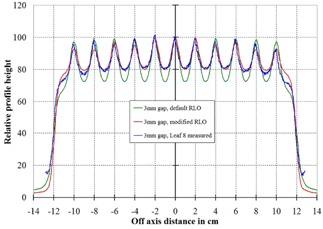
Relative dose profiles from the Pinnacle planar dose through leaf number 8, as shown in Fig. 5. These are for a 3 mm gap in the Picket Fence test. The red curve is the Pinnacle‐calculated profile using the modified RLO table, the green is using the default table. The blue curve shows the measured profile from the Picket Fence image.

**Figure 7:**
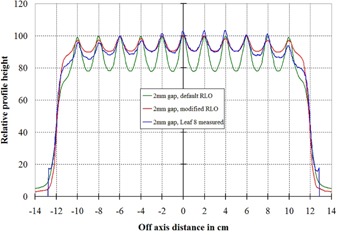
Relative dose profiles from the Pinnacle planar dose through leaf number 8, as shown in Fig. 5. These are for a 2 mm gap in the Picket Fence test. The red curve is the Pinnacle‐calculated profile using the modified RLO table, the green is using the default table. The blue curve shows the measured profile from the Picket Fence image.

To show that the modified RLO table more accurately modeled the actual measured data, a Picket Fence type test was performed, as described in the Methods section. The measured data from film were compared with the Pinnacle‐calculated values and are shown in Fig. 6 for the 3 mm gap and Fig. 7 for the 2 mm gap. In both cases, it can be seem that the modified RLO table better modeled the delivered dose.

### A. Light field‐based calibration

**Figure 8:**
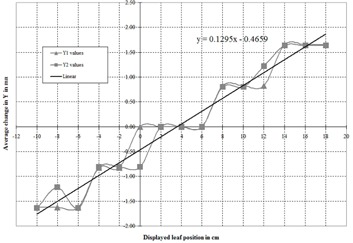
Correspondence between dosimetric‐based and light field‐based MLC calibrations. The straight line is a linear least squares fit to the average of Y1 and Y2 for each displayed leaf position. Fitting parameters are shown in the figure.

As described in the Methods section, conversion data were found between a dosimetric‐based and light field‐based calibration for the 80‐leaf Elekta Synergy. The conversion data are found as the fitted line in Fig. 8. These linear data were then combined with the modified RLO table shown as either the red or green curve of Fig. 1 to produce the purple curve of Fig. 1. The MLC leaves could then be calibrated using the light field and then, using the purple curve of Fig. 1 as the RLO table, obtain the same correct results as described above for the dosimetric based calibration.

## IV. CONCLUSIONS

A method based on calculated dose profiles was developed to obtain machine‐specific rounded leaf offsets in the Pinnacle^3^ treatment planning software. The method works directly if the user utilizes a dosimetric MLC leaf calibration, but can also work with a light field‐based calibration if the user implements the light field conversion method described in the Methods section. A Picket Fence test using both 2 and 3 mm gaps showed that the modified RLO tables better modeled the radiation field than the vendor supplied default tables.

It must be emphasized that the specific modified RLO data presented in this paper (see the red, green, and purple curves of Fig. 1) for the Elekta 80‐leaf MLC apply strictly only to the unique machine model used by the author. Readers should use the method described in the Methods section to determine their own model‐specific RLO curves and are, therefore, discouraged from simply using the modified RLO data presented in the Results section.

## Supporting information

Supplementary Material

Supplementary Material

Supplementary Material
