# Supplementary figures and images for: Optimization of the rounded leaf offset table in modeling the multileaf collimator leaf edge in a commercial treatment planning system
Source: J Appl Clin Med Phys. 2014 Nov 8;15(6):128–37. doi: 10.1120/jacmp.v15i6.4899 (PMC5711105; doi:10.1120/jacmp.v15i6.4899)

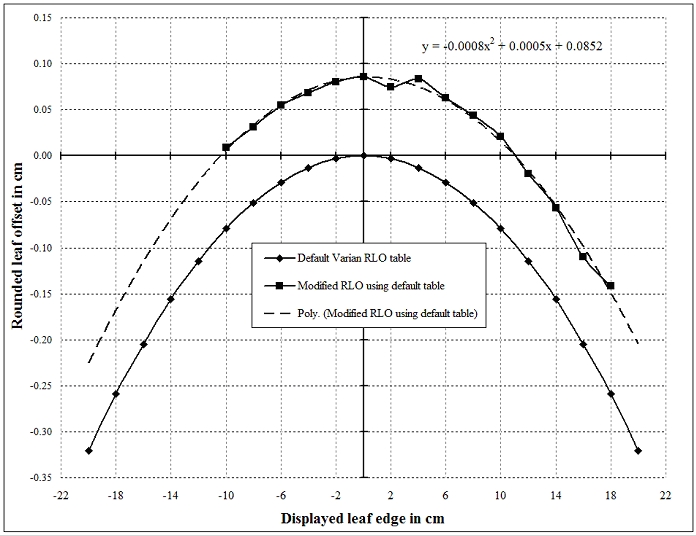

Supplement: Supplementary file 1 — Supplementary Material [file ACM2-15-128-s001.jpg]

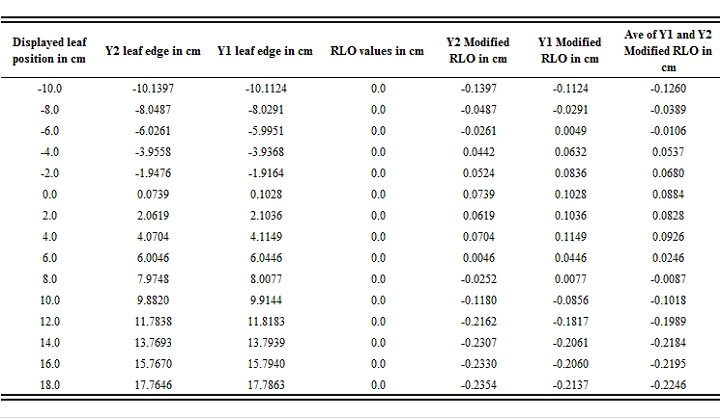

Supplement: Supplementary file 2 — Supplementary Material [file ACM2-15-128-s002.jpg]

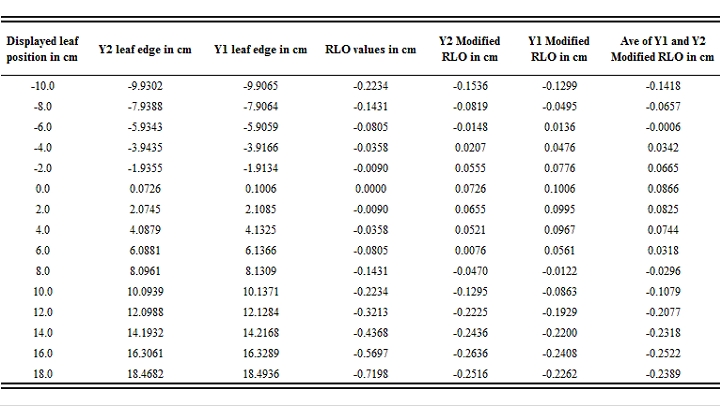

Supplement: Supplementary file 3 — Supplementary Material [file ACM2-15-128-s003.jpg]
